# Context sensitivity in the detection of changes in facial emotion

**DOI:** 10.1038/srep27798

**Published:** 2016-06-13

**Authors:** Yuichi Yamashita, Tomomi Fujimura, Kentaro Katahira, Manabu Honda, Masato Okada, Kazuo Okanoya

**Affiliations:** 1 Department of Functional Brain Research, National Institute of Neuroscience, National Center of Neurology and Psychiatry, 4-1-1 Ogawa-Higashi, Kodaira, Tokyo 187-8502, Japan; 2 Japan Science and Technology Agency, ERATO, Okanoya Emotional Information Project, 2-1 Hirosawa, Wako-shi, Saitama, 351-0198 Japan; 3 Behavior and Cognition Joint Research Laboratory, RIKEN Brain Science Institute, Wako, Saitama, Japan 2-1 Hirosawa, Wako-shi, Saitama, 351-0198 Japan; 4 Human Informatics Research Institute, National Institute of Advanced Industrial Science and Technology, AIST Central 2, 1-1-1 Umezono, Tsukuba, Ibaraki 305-8568, Japan; 5 Department of Psychology, Graduate School of Environmental Studies, Nagoya University, Furo-cho, Chikusa-ku, Nagoya 464-8601, Japan; 6 Department of Complexity Science and Engineering, Graduate School of Frontier Sciences, The University of Tokyo, 5-1-5 Kashiwanoha, Kashiwa, 277-8561 Chiba, Japan; 7 Department of Cognitive and Behavioral Sciences, Graduate School of Arts and Sciences, The University of Tokyo, 3-8-1 Komaba, Meguro-ku, Tokyo 153-8902, Japan

## Abstract

In social contexts, reading subtle changes in others’ facial expressions is a crucial communication skill. To measure this ability, we developed an expression-change detection task, wherein a series of pictures of changes in an individual’s facial expressions within contextual scenes were presented. The results demonstrated that the detection of subtle changes was highly sensitive to contextual stimuli. That is, participants identified the direction of facial-expression changes more accurately and more quickly when they were ‘appropriate’—consistent with the valence of the contextual stimulus change—than when they were ‘inappropriate’. Moreover, individual differences in sensitivity to contextual stimuli were correlated with scores on the Toronto Alexithymia Scale, a commonly used measure of alexithymia tendencies. These results suggest that the current behavioural task might facilitate investigations of the role of context in human social cognition.

Facial expressions that convey emotional information in communication is an important topic in the scientific study of emotion. In particular, extensive research in the area of emotion has been conducted on the recognition of facial expressions; that is, the identification and discrimination between emotions using facial expressions as cues^1^,^2^,^3^,^4^. Additionally, during actual communication, the meaning of facial expressions must be determined by considering the situational background/context.

Indeed, the relationship between the recognition of facial expressions and its background/context has been investigated extensively from diverse perspectives, using behavioural, physiological, and neuroimaging techniques. For example, the identification of emotions associated with facial expressions has been reported to be significantly affected by contextual inputs, which include body postures^5^,^6^,^7^, voices^8^,^9^, background scenes^10^,^11^, and words^12^,^13^. Moreover, the influence of context is inevitable and persists even when subjects are instructed to ignore such information^7^. However, because these studies focused on the integration of multi-modal sensory stimuli, the temporal aspect of context, i.e. the relationship between contextual information and the resulting changes in others’ emotional states, was considered as being outside their scope.

The temporal aspect of context in the recognition of emotion has been investigated extensively using a well-established experimental paradigm of mood-congruent/affective priming effects^14^,^15^,^16^,^17^. Affective priming is a phenomenon in that the evaluation of a positive or negative target preceded by a valence-congruent prime (i.e. positive prime–positive target or a negative prime–negative target) are faster and more accurate compared to an incongruent prime (i.e., positive prime–negative target or negative prime–positive target)^14^,^15^,^16^,^17^. According to the prevailing theory, affective priming effects emerge because the affective prime activates the memory representation of the affectively-related target, resulting in a faster and more accurate response to the target with the same emotional valence^14^,^15^,^17^.

Although such processes may play a crucial role in the integration of temporal context in emotion recognition, in everyday life, matching between a preceding stimulus and a following stimulus is not enough for the integration of temporal context in emotion recognition. For example, an individual might show a facial expression indicating extreme happiness upon receiving a gift, but after opening the wrapping, this expression might shift to indicate slight happiness. Even though the individual’s facial expression still can be identified as a positive emotion, the transition from very happy to slightly happy might suggest a negative shift in emotion: he/she might be a little disappointed by the gift. In such situations, rather than a valence, the direction of change in facial expression associated with a context has a crucial meaning. Indeed, recent studies have emphasised the importance of the change in direction of facial expression in emotion recognition^18^,^19^. These studies found that video-clips of dynamically changing facial expressions can induce the anticipatory process of facial expression change, and this anticipatory process might be involved in mindreading mechanisms, such as theory of mind^19^.

In addition to the ability to read ‘changes’ in others’ facial expressions, the meanings of changes in facial expression must be determined by integrating the situational background/context. Moreover, the critical role of background/context information in social communication implies that impaired recognition of emotion, resulting from poor integration of contextual information might be related to mental disorders, including autism spectrum disorders (ASD) and alexithymia, which involve impairments in describing and identifying emotions^20^,^21^.

However, an experimental paradigm to investigate the mechanism underlying the integration of contextual information in the recognition of changes in facial expressions has not yet been fully explored. In order to address this issue, the current study aimed to develop a novel behavioural task to measure the effect of contextual information on the recognition of changes in facial expression. Additionally, we investigated the relationship between the ability to identify/describe emotions and individual differences in emotion recognition with contextual information.

## Methods

### Participants

The participants consisted of twenty university students (10 females and 10 males age: M ± SD = 20.0 ± 3.5 years). All participants were native Japanese speakers, with normal or correct-to-normal eyesight, and no history of mental or neurological illness. Prior to their enrolment in the study, all participants signed an informed consent form. All of the experiments were carried out in accordance with the guidelines and regulations of Japan’s Ministry of Health, Labour and Welfare. The protocol used to collect and analyse the data was approved by the University of Tokyo Ethics Committee (#231-2).

### Stimulus materials and task procedure

**Figure 1:**
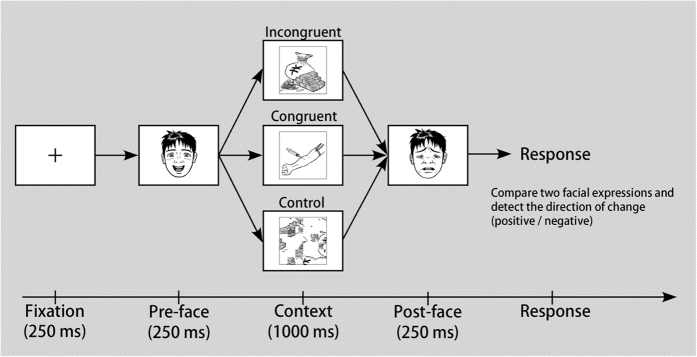
Schematic of the trial sequences and the three experimental conditions. Sample pictures of facial and emotional-context images were used in this figure to avoid using images from the ATR facial expression database and IAPS, which were used in the study. Permission to use of the drawings in this figure was obtained from Taiki Kobayashi for publication under an Open Access license.

Figure 1 depicts the task design. In each trial, a central fixation cross was presented for 500 ms, followed by successive presentations of a pre-face (250 ms), a context scene (1000 ms), and a post-face (250 ms), each with a 250-ms interval. Participants were required to identify quickly and accurately, the direction (positive/negative) of facial expression changes between the pre- and post-faces, by pressing a button (e.g. the left button for a negative change and right button for a positive change).

The faces of eight individuals depicting emotions (4 male and 4 female Japanese models), from the Advanced Telecommunications Research (ATR) facial expression database^22^, were used as facial stimuli. The ATR facial expression database provides a set of facial-expression photos of Japanese models as experimental stimuli for academic studies. The quality of the images and the intensity of each type of facial expression has been standardised and clarified through psychological evaluations^22^. Continua of facial expressions with changes in emotional valence were created using a morphing software program^23^. Prototypical facial expressions of happy and fearful faces were selected as the endpoints of the facial expressions continuum to represent emotional valence-based continua. A fearful face was used because a fearful expression is more suitable as a response to the contents of a scene with a negative context (see below) rather than a sad or angry expression. In addition, morphed images of fearful and happy expressions were used in several studies to represent valence-based continua with similar arousal levels^1^,^2^,^3^,^4^. For each model, facial images were morphed into seven in-between steps from happy to fearful. Of these, six images (besides the faces at both ends) were used as task stimuli and were referred to as ‘face-1’–‘face-6’. The degree of physical change in the faces in the continua were similar to those used in the discrimination tasks in previous studies^3^,^4^. Eight continua of facial expressions were created using prototypical facial expressions of the eight models. This served to diversify the features in the stimulus set. Based on the brightness distributions, we confirmed that low-level visual features were similar among the morphed facial images (Supplementary TableS1).

Pairs of faces (pre- and post-faces), one or two steps apart on the continuum (e.g. from face-1 to face-3, from face-5 to face-4, etc.) were selected to produce four conditions of physical distance between the facial pairs (from −2 steps to +2 steps apart on the continuum) as the ‘distance’ variable. Two conditions of the ‘category’ variable were used: pairs of faces between the centre of the continuum (between face-3 and face-4) were considered cross-category pairs, and those from the periphery of the facial expression continuum were considered as within-category pairs.

Contextual scenes were inserted between the two facial images. Scene images were selected from the International Affective Picture System (IAPS)^24^. From the IAPS, ten pleasant and ten unpleasant scenes were selected, such as images of a lot of money and fireworks for positive images, and images of an injection and a traffic accident for negative images. The average valence and arousal levels of the positive images were 7.73 ± 0.26 and 5.09 ± 0.75, and the average valence and arousal levels of negative images were 2.64 ± 0.55 and 6.07 ± 0.66. The full list of the slide numbers and detailed descriptions used in the current study are presented in Supplementary Table S2. For the control condition, each set of affective images were shuffled to ensure that the images were meaningless.

Based on the combinations between the direction of changes in facial expression (positive/negative) and emotional valence of the inserted contextual images, three conditions of the ‘congruency’ variable were used. (1) In the congruent condition, the direction of the facial expression change and valence of the contextual image were consistent. The relationship between the facial expression change and contextual information matched those observed in everyday life (‘appropriate’ facial expression changes). (2) In the incongruent condition, the direction of changes and valence of the inserted image were inconsistent (‘inappropriate’ facial expression changes). (3) The control condition included the shuffled meaningless images. However, congruency differed from the usual associations in the semantic/emotional-priming paradigm: for example, in the current study, a transition from very happy to slightly happy was a negative shift even though both faces’ emotional valences were positive, implying that its association with a negative scene was congruent.

As a result, there were 24 (3 ‘congruency’ × 2 ‘category’ × 4 ‘distance’) conditions. The experiment consisted of 240 trials separated into 5 blocks, in which each condition appeared twice. The order of condition for each task was pseudorandomised, and facial models and contextual scenes were randomly selected from the stimulus pool (10 positive, 10 negative, and 20 meaningless images).

### Data analysis

Although morphed images of the facial expressions were used, the participants task was to detect the direction of the changes from the first facial image to the second facial image. Thus, the correct answer for each trial was explicitly defined. The mean values of the rate of correct responses and the median reaction time (RT) per participant were used in the data analyses. Repeated-measures ANOVAs were conducted for congruency, category, and distance variables. All post hoc multiple comparisons were performed using Shaffer’s modified sequentially rejective Bonferroni procedure^25^. The level of statistical significance was set at *p* < *0.05*.

## Results

### Rate of correct responses

**Figure 2:**
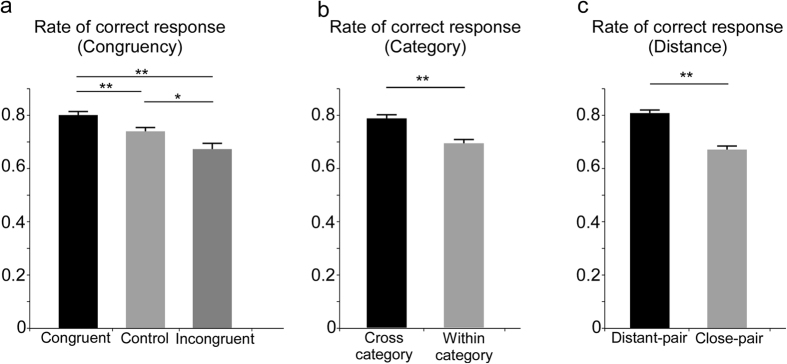
Rates of correct responses in each condition for (**a**) congruency, (**b**) category, and (**c**) distance. Error bars indicate standard errors. * and ** indicate statistical significance of p < 0.05 and p < 0.01, respectively.

The 3 × 2 × 4 repeated-measures ANOVA on the mean correct response rate revealed a significant main effect of congruency (*F (2, 38)* = *11.8, p* < *0.01*), category (*F (1, 19)* = *39.77, p* < *0.01*), and distance (*F (1, 19)* = *123.28, p* < *0.01*). Regarding the main effect of congruency, multiple comparisons revealed that the rate of correct responses was significantly higher in the congruent condition compared to the control (*p* < *0.01*) and incongruent (*p* < *0.01*) conditions, and it was significantly lower in the incongruent condition (*p* < *0.01*) than in the control condition (Fig. 2a), indicating that the detection of subtle changes in facial expressions was highly sensitive to contextual stimuli.

Regarding the main effect of category, the correct response rate was significantly higher in the cross-category condition than the within-category condition (Fig. 2b), suggesting that changes in facial expressions were perceived more precisely when the facial pairs were selected across emotion categories than from the ends of the facial expression continuum, even though the degree of physical changes of both were assumed to be identical. In addition, the main effect of distance indicated that the correct response rate was significantly higher in the distant-pair condition than in the close-pair condition (Fig. 2c), demonstrating that larger changes in facial expression were easier to detect than smaller changes.

**Figure 3:**
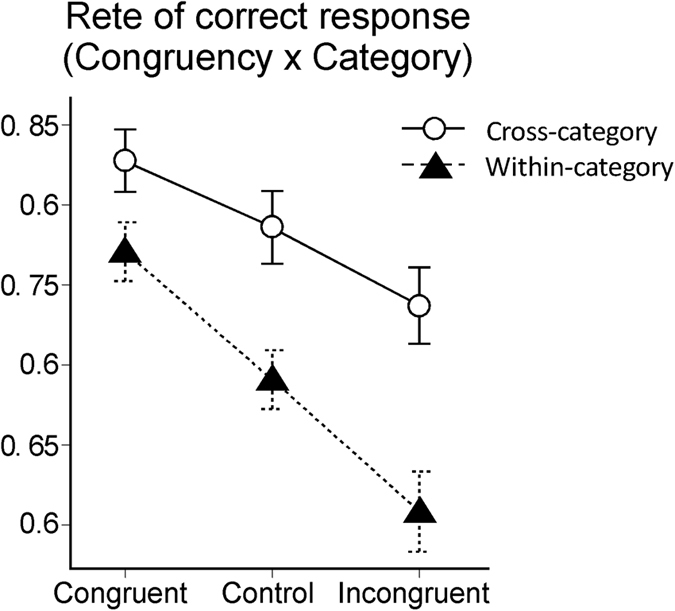
Rates of correct responses in each condition for congruency and category. Error bars indicate standard errors.

The repeated-measures ANOVA also yielded a significant interaction between congruency and category (*F (2, 38)* = *4.17, p* < *0.05*) and a marginally significant interaction between congruency, category, and distance (*F (2, 38)* = *2.77, p* < *0.10*). To investigate the significance of the interaction between congruency and category, simple main effects analyses were performed. A significant simple main effect of category type on the congruent condition (*F (1, 19)* = *8.17, p* < *0.05*), control condition (*F (1, 19)* = *23.46, p* < *0.01*), and incongruent condition (*F (1, 19)* = *33.31, p* < *0.01*) were found. In addition, there was a significant simple main effect of congruency type on the cross-category condition (*F (2, 38)* = *5.25, p* < *0.01*) and within category condition (*F (2, 38)* = *14.73, p* < *0.01*). Multiple comparisons of the effect of congruency type in the cross-category condition showed no significant differences among the congruent, control, and incongruent conditions. However, in the within-category condition, the rate of correct responses was significantly higher in the congruent condition compared to the control (*p* < *0.01*) and incongruent conditions (*p* < *0.01*), and it was significantly lower in the incongruent condition than in the control condition (*p* < *0.01*) (Fig. 3).

These results indicated that the congruency effect was diminished in the cross-category condition compared to the within-category condition. However, this difference was difficult to distinguish from the influence of task difficulty, depending on the category type. Thus, the significant interaction between congruency and category may be attributable to the ceiling effect in the cross-category condition (the relatively easier condition).

### Reaction time (RT)

**Figure 4:**
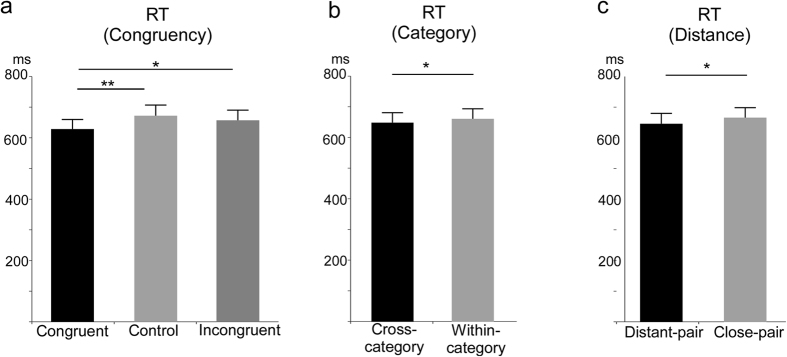
Mean reaction time in each condition for (**a**) congruency, (**b**) category, and (**c**) distance. Error bars indicate standard errors. * and ** indicate statistical significance of p < 0.05 and p < 0.01, respectively.

The 3 × 2 × 4 repeated-measures ANOVA of the mean RTs revealed a significant main effect of congruency (*F (2, 38)* = *10.63, p* < *0.01*), category (*F (1, 19)* = *5.21, p* < *0.05*), and distance (*F (1, 19)* = *7.80, p* < *0.05*). Regarding the main effect of congruency, multiple comparisons revealed that the RT was significantly shorter in the congruent condition compared to the control (*p* < *0.01*) and incongruent (*p* < *0.05*) conditions (Fig. 4a). Similar to the rate of correct responses, the RT showed that the detection of subtle changes in facial expressions was highly sensitive to context.

Regarding the main effect of category, the RT was significantly shorter in the cross-category condition than in the within-category condition (Fig. 4b). In addition, the main effect of distance indicated that the RT was significantly shorter in the distant-pair condition than in the close-pair condition (Fig. 4c). These observations consistently indicated that changes in facial expression were perceived more quickly when the facial pairs were selected among the emotion categories than from the ends of the facial expression continuum, and that larger changes in facial expression were more quickly detected than were smaller changes.

**Figure 5:**
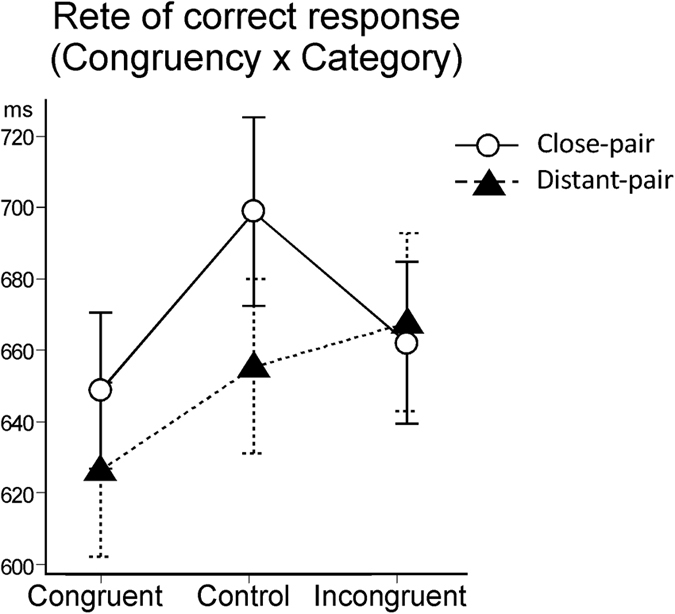
Mean reaction time in each condition for congruency and distance. Error bars indicate standard errors.

The repeated-measures ANOVA also yielded a significant interaction between congruency and distance (*F (2, 38)* = *10.63, p* < *0.01*) and a marginally significant interaction between congruency and category (*F (2, 38)* = *2.81, p* < *0.10*). To investigate the significance of the interaction between congruency and distance, simple main effects analyses were performed. The analyses showed a significant simple main effect of distance type on the control condition (*F (1, 19)* = *11.72, p* < *0.01*). There were no significant simple main effects of distance on the congruent and incongruent conditions. However, there was a significant simple main effect of congruency type on the close-pair condition (*F (2, 38)* = *12.08, p* < *0.01*) and distant-pair condition (*F (2, 38)* = *5.73, p* < *0.01*). Multiple comparisons of the effect of congruency type in the close-pair condition found that the RT was significantly longer in the control condition compared to the congruent (*p* < *0.01*) and incongruent (*p* < *0.01*) conditions. In the distant-pair condition, however, the RT was significantly shorter in the congruent condition compared to the control (*p* < *0.05*) and incongruent (*p* < *0.05*) conditions (Fig. 5).

### Ability to identify/describe emotions

We also investigated the relationship between individual differences in the ability to identify/describe emotions and emotion recognition with contextual information. The ability to identify/describe emotions was evaluated using the Toronto Alexithymia Scale (TAS-20), a commonly used measure of alexithymia tendency^26^. As measures of individual differences in sensitivity to contextual stimuli, the differences in the proportion correct between the congruent and incongruent conditions (congruency effect) on the within-category condition was tested. This analysis was based on the finding that the effect of context was diminished in the cross-category condition. In addition, based on the possibility of a ceiling effect in the cross-category trial, we assumed that the congruency effect in the most difficult conditions might be a more accurate indicator of individual differences. Based on this assumption, we also tested the congruency effect on the within-category and close-pair trials (assumed to be the most difficult conditions) as measures of individual differences in sensitivity to contextual stimuli.

**Figure 6:**
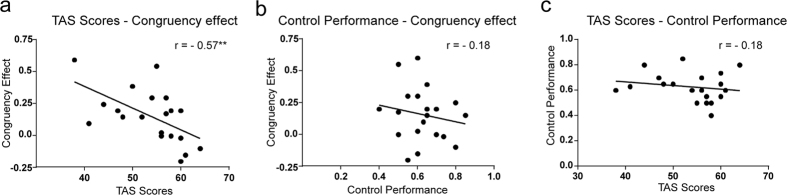
Scatter plots and correlation coefficients for the function of TAS scores and task performance for each of the following relationships. (**a**) TAS scores and the congruency effect, (**b**) performance in the control condition (shuffled, meaningless contextual images) and congruency effect, (**c**) TAS scores and performance in the control condition. In all plots, the congruency effect was calculated as the difference in the proportion correct between the congruent and incongruent conditions on the within-category and close-pair conditions. **Indicates statistical significance of *p* < *0.01*.

The congruency effect on the within-category condition showed a marginally significant negative correlation with the TAS scores (*r* = −*0.41, p* < *0.1*; Supplementary Figure S1a); however, the congruency effect on the within-category and close-pair conditions showed a significant negative correlation with the TAS scores (*r* = −*0.57, p* < *0.01*; Fig. 6a). On the other hand, the congruency effect and the performance of the control (without context) condition showed no significant correlation (Fig. 6b), and the TAS scores were not significantly correlated with task performance in the control condition (Fig. 6c), in the within-category condition and in the within-category and close-pair condition (Supplementary Figures S1b and S1c).

## Discussion

This study found that the detection of subtle changes in facial expression was highly sensitive to contextual stimuli. That is, participants recognised subtle changes in facial expression more accurately and more quickly when the direction of the change was consistent with the valence of the contextual stimulus (‘appropriate’ emotional change), rather than inconsistent with the valence (‘inappropriate’ change in facial expression). In addition, individual differences in the sensitivity to contextual stimuli were correlated with participants’ TAS-20 scores, suggesting that the integration of contextual information in emotion recognition might play an important role in alexithymia. These results suggest that the behavioural task in this study was capable of measuring the effect of contextual information on the recognition of changes in facial expressions, implying that it could be useful in studies on human social cognition.

One may argue that the current study’s observed effect of contextual information on the recognition of changes in facial expressions was equivalent to a simple mood-congruent/affective priming effect^14^,^15^,^16^,^17^ of contextual images. However, there was an important difference between this study’s observations and simple affective priming phenomenon. In simple affective priming with facial expression recognition, evaluation of a target face is enhanced when the valence of the preceding prime (corresponding to the contextual scene in the current study’s task) match the valence of the target facial expression. However, in the current study’s task, an increased rate of correct responses and faster RTs were observed even in the condition in which the valence of the contextual scene did not match the valence of the following facial stimulus (post-face). This finding is due to the use of a definition of congruency in the current study, which differed from that used in the usual affective priming paradigm. For example, a transition from very happy to slightly happy was defined as a negative shift even though the emotional valences of both faces’ were positive, implying that the association with a negative scene was congruent. Therefore, the current findings suggest that the direction of changes, rather than the facial expression itself, have a positive/negative valence.

This idea is consistent with previous findings, wherein the recognition of a neutral face was affected by preceding clips of changes in facial expressions^18^,^19^. In these studies, an initial positive or negative facial expression gradually morphed into a neutral expression; the recognition of a neutral facial image displayed at the end of the short video-clips were perceived as indicative of valence, rather than neutrality, suggesting that the direction of change had a positive/negative valence. The authors of these studies claim that this overshoot in the recognition of dynamic facial expressions was not caused by low-level perceptual mechanisms (e.g. adaptation and representational momentum), but by a higher-level process of ‘emotional anticipation’ (a low-level mindreading mechanism)^19^.

The current study’s findings of a faster RT in the congruent condition and a slower RT in the incongruent condition suggest that the participants might have automatically anticipated ‘appropriate’ changes in the emotional state associated with the context, even though the context image should have been ignored to solve the task. Such context-induced anticipation of emotional state transition might be related to involuntary simulation processes of other’s mental state, referred to as emotional anticipation or low-level mindreading^19^,^27^. However, to understand the underlying mechanisms and the relationship to those processes, further investigations are needed. For example, the present study’s task instruction requiring detection of the direction of change might have affected the observed phenomenon. Even in behavioural tasks involving automatic unconscious processes, such as emotional priming, task instructions affect observations of phenomena^17^. Therefore, an investigation of whether similar observations can be obtained using the same stimulus set with implicit task settings should provide useful information.

In the present study, changes in facial expression were perceived more accurately for facial pairs across emotion categories compared to those from the ends of the facial-expression continuum, even though the degree of physical changes of both pairs were assumed to be identical. This finding suggests that changes in facial expressions were perceived as a continuous change of physical features as well as a categorical: as a transition of stereotypical, discrete emotion states. This is consistent with the well-established theory that facial-expression recognition reflects a categorical perception, which is involved in higher cognitive functions, such as labelling and symbol manipulations^13^,^28^,^29^. Such functions, including labelling or symbol manipulation might affect the observed interaction between congruency and category. That is, in the cross-category condition, facial expression changes were perceived more explicitly as a discrete state transition using labelling or symbol manipulation, resulting in the reduced impact of contextual stimuli, which should be ignored to solve the task. However, category perception might also result in the reduction of task difficulty. Therefore, the seemingly reduced congruency effect in the cross-category condition might be attributable to a ceiling effect.

In the RT analysis, a significant interaction between congruency and distance was found. In the distant-pair condition, RT was significantly shorter in the congruent condition compared to the control and incongruent conditions, suggesting that the difference in RT may reflect sensitivity to context. However, in the close-pair condition, the RT was significantly longer in the in the control condition compared to the congruent and incongruent conditions, implying that the difference in RT might reflect the level of arousal induced by the contextual images with emotional content. These findings might have resulted from the complicated relationship among the task’s difficulty, the congruency effect of context, and the arousal induced by emotional stimuli. Therefore, in the current form of the task, the rate of correct responses seems to be a more suitable measure for evaluating the effect of context in the recognition of changes in facial expression. Further studies are needed to distinguish these effects.

Further, the analysis revealed that individual differences in the sensitivity to context stimuli (congruency effect) in the within-category and close-pair condition was negatively correlated with TAS-20 scores; individuals with higher sensitivity to context were less likely to have alexithymia tendencies, which is a personality trait characterised by the inability to identify and describe one’s own emotions^21^,^30^. However, this correlation was diminished in the within-category condition, suggesting that individual differences in the congruency effect might have been more accurately reflected under the most difficult conditions. On the other hand, sensitivity to context was not correlated with the ability to detect changes in facial expression (performance in the control condition), which suggests that the integration of contextual information in emotion recognition may play an important role in alexithymia. This is consistent with the finding that individuals with alexithymia exhibit emotional responses that are ineffective and lack empathy^21^ and high rates of overlap with ASD symptoms, suggesting impairments related to the theory of mind^20^. Collectively, the findings suggest that the task provides a quantitative measure of the effect of contextual information on emotion recognition that can be used to investigate this characteristic feature of human social cognition and its role in alexithymia and ASD.

## Additional Information

**How to cite this article**: Yamashita, Y. *et al*. Context sensitivity in the detection of changes in facial emotion. *Sci. Rep.* **6**, 27798; doi: 10.1038/srep27798 (2016).

## Supplementary Material

Supplementary Information

## References

[b1] Calder A. J., Young A. W., Perrett D. I., Etcoff N. L. & Rowland D. Categorical perception of morphed facial expressions. Vis. Cogn. 3, 81–118, 10.1080/713756735 (1996).

[b2] Young A. W. . Facial expression megamix: Tests of dimensional and category accounts of emotion recognition. Cognition 63, 271–313, 10.1016/S0010-0277(97)00003-6 (1997). 9265872

[b3] Fujimura T., Matsuda Y. T., Katahira K., Okada M. & Okanoya K. Categorical and dimensional perceptions in decoding emotional facial expressions. Cogn. Emot. 26, 587–601, 10.1080/02699931.2011.595391 (2012). 21824015 PMC3379784

[b4] Maeshima H., Yamashita Y., Fujimura T., Okada M. & Okanoya K. Modulation of emotional category induced by temporal factors in emotion recognition. PLoS One 10, 10.1371/journal.pone.0131636 (2015). PMC4521787 26230992

[b5] Meeren H. K., van Heijnsbergen C. C. & de Gelder B. Rapid perceptual integration of facial expression and emotional body language. Proc. Natl. Acad. Sci. USA 102, 16518–16523, 10.1073/pnas.0507650102 (2005). 16260734 PMC1283446

[b6] Aviezer H. . Angry, disgusted or afraid? Studies on the malleability of emotion perception. Psychol. Sci. 19, 724–732, 10.1111/j.1467-9280.2008.02148.x (2008). 18727789

[b7] Aviezer H., Trope Y. & Todorov A. Body cues, not facial expressions, discriminate between intense positive and negative emotions. Science. 338, 1225–1229, 10.1126/science.1224313 (2012). 23197536

[b8] de Gelder B., Böcker K. B., Tuomainen J., Hensen M. & Vroomen J. The combined perception of emotion from voice and face: Early interaction revealed by human electric brain responses. Neurosci. Let. 260, 133–136, 10.1016/S0304-3940(98)00963-X (1999). 10025717

[b9] de Gelder B. & Vroomen J. The perception of emotions by ear and by eye. Cogn. Emot. 14, 289–311, 10.1080/026999300378824 (2000).

[b10] Righart R. & De Gelder B. Recognition of facial expressions is influenced by emotion scene gist. Cogn. Affect. Behav. Neurosci. 8, 264–278, 10.3758/CABN.8.3.264 (2008). 18814463

[b11] Barrett L. F. & Kensinger E. A. Context is routinely encoded during emotion perception. Psychol Sci. 21, 595–599, 10.1177/0956797610363547 (2010). 20424107 PMC2878776

[b12] Lindquist K. A., Barrett L. F., Bliss-Moreau E. & Russell J. A. Language and the perception of emotion. Emotion 6, 125–138, 10.1016/j.tics.2007.06.003 (2006). 16637756

[b13] Barrett L. F., Lindquist K. A. & Gendron M. Language as context for the perception of emotion. Trends Cogn. Sci. 11, 327–332, 10.1016/j.tics.2007.06.003 (2007). 17625952 PMC2225544

[b14] Fazio R. H. On the automatic activation of associated evaluations: an overview. Cogn. Emot. 15, 115–141, 10.1080/0269993004200024 (2001).

[b15] Klauer K. C. & Musch J. Affective priming: Findings and theories. In *The Psychology of Evaluation: Affective* Processes *in Cognition and Emotion*. (eds Musch, J. & Klauer, K. C.), 7–51 (Lawrence Erlbaum Associates, 2003).

[b16] Carroll N. C. & Young A. W., Priming of emotion recognition. Q. J. Exp. Psychol. 58A, 1173–1197, 10.1080/02724980443000539 (2005). 16194954

[b17] Spruyt A., De Houwer J., Hermans D. & Eelen P. Affective priming of nonaffective semantic categorization responses. Experimental Psychology 54, 44–53, 10.1080/02699930143000419 (2007). 17341014

[b18] Jellema T., Pecchinenda A., Palumbo L. & Tan E. G. Biases in the perception and affective valence of neutral facial expressions induced by the immediate perceptual history. Vis. Cogn. 19, 616–634, 10.1080/13506285.2011.569775 (2011).

[b19] Palumbo L. & Jellema T. Beyond face value: does involuntary emotional anticipation shape the perception of dynamic facial expressions? PLoS One 8, 10.1371/journal.pone.0056003 (2013). PMC3569428 23409112

[b20] Hill E., Berthoz S. & Frith U. Brief report: cognitive processing of own emotions in individuals with autistic spectrum disorder and in their relatives. J. Autism Dev. Disord. 34, 229–23, 10.1023/B:JADD.0000022613.41399.14 (2004). 15162941

[b21] Feldmanhall O., Dalgleish T. & Mobbs D. Alexithymia decreases altruism in real social decisions. Cortex 49, 899–904, 10.1016/j.cortex.2012.10.015 (2012). 23245426

[b22] AdvancedTeleommunications Research-Promotions. A. T. R. Database of facial expression DB99, ATR-Promotions. (2006) Available at: http://www.atr-p.com/products/pdf/face-db(J).pdf. (Accessed: 15th April 2016).

[b23] Sqirlz Morph. Available at: http://www.xiberpix.net/SqirlzMorph.html. (Accessed: 15th April 2016).

[b24] Land P. J., Bradley M. M. & Cuthbert B. N. International Affective Picture System (IAPS): Affective Ratings of Pictures and Instruction Manual. Technical Report A-8. (University of Florida, 2008).

[b25] Shaffer J. P. Modified sequentially rejective multiple test procedures. J. Am. Stat. Assoc. 81, 826–831, 10.2307/2289016 (1986).

[b26] Bagby R. M., Parker J. D. A. & Taylor G. J. The twenty-item Toronto Alexithymia Scale-I. Item selection and cross-validation of the factor structure. J. Psychosom Res. 38, 23–32, 10.1016/0022-3999(94)90005-1 (1994). 8126686

[b27] Goldman A. I. & Sripada C. S. Simulationist models of face-based emotion recognition. Cognition 94, 193–213, 10.1016/j.cognition.2004.01.005 (2005). 15617671

[b28] Roberson D. & Davidoff J. The categorical perception of colors and facial expressions: the effect of verbal interference. Mem. Cogn. 28, 977–986, 10.3758/BF03209345 (2000). 11105523

[b29] Roberson D., Damjanovic L. & Pilling M. Categorical perception of facial expressions: evidence for a “category adjustment” model. Mem. Cogn. 35, 1814–1829, 10.3758/BF03193512 (2007). 18062556

[b30] Sifneos P. E. The prevalence of ‘alexithymic’ characteristics in psychosomatic patients. Psychother. Psychosom. 22, 255–262, 10.1159/000286529 (1973). 4770536

