# Supplementary material for: Context sensitivity in the detection of changes in facial emotion
Source: Sci Rep. 2016 Jun 13;6:27798. doi: 10.1038/srep27798 (PMC4904217; doi:10.1038/srep27798)
Supplement: Supplementary Information [file srep27798-s1.doc]

**Supplemental information**

**Context sensitivity in the detection of changes in facial emotion**

Yuichi Yamashita, Tomomi Fujimura, Kentaro Katahira, Manabu Honda, Masato Okada and Kazuo Okanoya

| Model | Negative | | | | Positive | | | |
| --- | --- | --- | --- | --- | --- | --- | --- | --- |
|  | Mean | SD | Skewness | Kurtosis | Mean | SD | Skewness | Kurtosis |
| ID1 | 0.30 | 0.17 | 0.19 | -1.40 | 0.29 | 0.17 | 0.34 | -1.35 |
| ID2 | 0.27 | 0.15 | 0.31 | -1.30 | 0.29 | 0.16 | 0.28 | -1.34 |
| ID3 | 0.27 | 0.15 | 0.09 | -1.40 | 0.29 | 0.14 | 0.09 | -1.45 |
| ID4 | 0.32 | 0.14 | -0.31 | -1.25 | 0.34 | 0.15 | -0.32 | -1.23 |
| ID5 | 0.32 | 0.14 | -0.28 | -1.11 | 0.32 | 0.15 | -0.29 | -1.06 |
| ID6 | 0.31 | 0.13 | -0.32 | -1.08 | 0.34 | 0.15 | -0.32 | -1.03 |
| ID7 | 0.32 | 0.16 | -0.27 | -1.29 | 0.33 | 0.17 | -0.22 | -1.34 |
| ID8 | 0.31 | 0.14 | -0.22 | -1.22 | 0.32 | 0.15 | -0.19 | -1.23 |

Supplementary Table S1. Examples of the brightness distributions of the morphed facial images

|  | Description | Slide | Valence | SD | Arousal | SD |
| --- | --- | --- | --- | --- | --- | --- |
| Positive | Porpoise | 1920 | 7.9 | 1.48 | 4.27 | 2.53 |
|  | Girls | 2091 | 7.68 | 1.43 | 4.51 | 2.28 |
|  | Family | 2340 | 8.03 | 1.26 | 4.9 | 2.2 |
|  | Children | 2345 | 7.41 | 1.72 | 5.42 | 2.47 |
|  | Children | 2347 | 7.83 | 1.36 | 5.56 | 2.34 |
|  | Couple | 2530 | 7.8 | 1.55 | 3.99 | 2.11 |
|  | Romance | 4614 | 7.15 | 1.44 | 4.67 | 2.47 |
|  | Fireworks | 5910 | 7.8 | 1.23 | 5.59 | 2.55 |
|  | Tubing | 8420 | 7.76 | 1.55 | 5.56 | 2.38 |
|  | Money | 8501 | 7.91 | 1.66 | 6.44 | 2.29 |
|  | Mean (positive) |  | 7.73 |  | 5.09 |  |
| Negative | Attack | 6550 | 2.73 | 2.38 | 7.09 | 1.98 |
|  | Suicide | 6570 | 2.19 | 1.72 | 6.24 | 2.16 |
|  | Gang | 6821 | 2.38 | 1.72 | 6.29 | 2.02 |
|  | Fire | 8485 | 2.73 | 1.62 | 6.46 | 2.1 |
|  | Execution | 9414 | 2.06 | 1.48 | 6.49 | 2.26 |
|  | Assault | 9425 | 2.67 | 1.44 | 5.92 | 2.13 |
|  | Dental Exam | 9584 | 3.34 | 1.57 | 4.96 | 2.15 |
|  | Injection | 9594 | 3.76 | 1.7 | 5.17 | 2.17 |
|  | Car Accident | 9900 | 2.46 | 1.39 | 5.58 | 2.13 |
|  | Fire | 9921 | 2.04 | 1.47 | 6.52 | 1.94 |
|  | Mean (negative) |  | 2.64 |  | 6.07 |  |

Supplementary Table S2. Detailed descriptions of IAPS images used in the current experiment


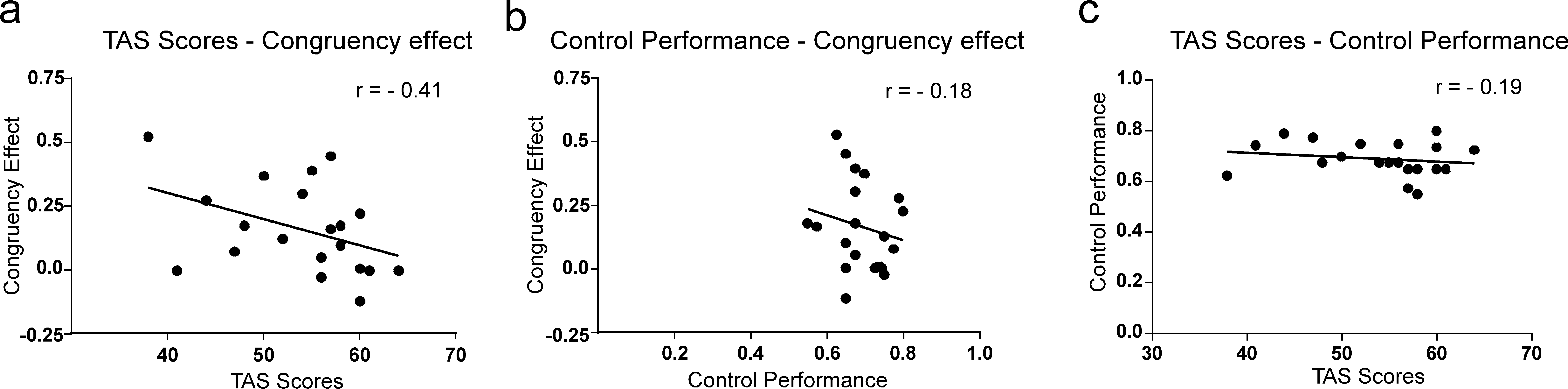


Supplementary Figure S1. Scatter plots and correlation coefficients for the function of TAS scores and task performance for each of the following relationships: (a) TAS scores and the congruency effect, (b) performance in the control condition (shuffled, meaningless contextual images) and congruency effect, (c) TAS scores and performance in the control condition. In all plots, the congruency effect was calculated as the difference in the proportion correct between the congruent and incongruent conditions on the within-category conditions.
